# Prediction of Glucose Intolerance in Early Postpartum in Women with Gestational Diabetes Mellitus Based on the 2013 WHO Criteria

**DOI:** 10.3390/jcm8030383

**Published:** 2019-03-19

**Authors:** Katrien Benhalima, Paul Van Crombrugge, Carolien Moyson, Johan Verhaeghe, Sofie Vandeginste, Hilde Verlaenen, Chris Vercammen, Toon Maes, Els Dufraimont, Christophe De Block, Yves Jacquemyn, Farah Mekahli, Katrien De Clippel, Annick Van Den Bruel, Anne Loccufier, Annouschka Laenen, Caro Minschart, Roland Devlieger, Chantal Mathieu

**Affiliations:** 1 Department of Endocrinology, University Hospital Gasthuisberg, KU Leuven, Herestraat 49, 3000 Leuven, Belgium; carolien.moyson@uzleuven.be (C.M.); caro.minschart@kuleuven.be (C.M.); chantal.mathieu@uzleuven.be (C.M.); 2 Department of Endocrinology, OLV Ziekenhuis Aalst-Asse-Ninove, Moorselbaan 164, 9300 Aalst, Belgium; Paul.Van.Crombrugge@olvz-aalst.be; 3 Department of Obstetrics & Gynecology, University Hospital Gasthuisberg, KU Leuven, Herestraat 49, 3000 Leuven, Belgium; johan.verhaeghe@uzleuven.be (J.V.); roland.devlieger@uzleuven.be (R.D.); 4 Department of Obstetrics & Gynecology, OLV Ziekenhuis Aalst-Asse-Ninove, Moorselbaan 164, 9300 Aalst, Belgium; Sofie.Vandeginste@olvz-aalst.be (S.V.); Hilde.Verlaenen@olvz-aalst.be (H.V.); 5 Department of Endocrinology, Imelda Ziekenhuis, Imeldalaan 9, 2820 Bonheiden, Belgium; Chris.Vercammen@imelda.be (C.V.); Toon.Maes@imelda.be (T.M.); 6 Department of Obstetrics & Gynecology, Imelda Ziekenhuis, Imeldalaan 9, 2820 Bonheiden, Belgium; Els.Dufraimont@imelda.be; 7 Department of Endocrinology-Diabetology-Metabolism, Antwerp University Hospital, Wilrijkstraat 10, 2560 Edegem, Belgium; Christophe.DeBlock@uza.be; 8 Department of Obstetrics & Gynecology, Antwerp University Hospital, Wilrijkstraat 10, 2560 Edegem, Belgium; Yves.Jacquemyn@uza.be; 9 Department of Endocrinology, Kliniek St-Jan Brussel, Kruidtuinlaan 32, 1000 Brussel, Belgium; fmekahli@clstjean.be; 10 Department of Obstetrics & Gynecology, Kliniek St-Jan Brussel, Kruidtuinlaan 32, 1000 Brussel, Belgium; kdeclippel@gmail.com; 11 Department of Endocrinology, AZ St Jan Brugge, Ruddershove 10, 8000 Brugge, Belgium; Annick.VandenBruel@azsintjan.be; 12 Department of Obstetrics & Gynecology, AZ St Jan Brugge, Ruddershove 10, 8000 Brugge, Belgium; anne.loccufier@azsintjan.be; 13 Center of Biostatics and Statistical bioinformatics, KU Leuven, Kapucijnenvoer 35 bloc d—box 7001, 3000 Leuven, Belgium; annouschka.laenen@kuleuven.be

**Keywords:** glucose intolerance, postpartum, gestational diabetes mellitus, 2013 WHO criteria, risk factors, prediction

## Abstract

Predictors for glucose intolerance postpartum were evaluated in women with gestational diabetes mellitus (GDM) based on the 2013 World Health Organization (WHO) criteria. 1841 women were tested for GDM in a prospective cohort study. A postpartum 75g oral glucose tolerance test (OGTT) was performed in women with GDM at 14 ± 4.1 weeks. Of all 231 mothers with GDM, 83.1% (192) had a postpartum OGTT of which 18.2% (35) had glucose intolerance. Women with glucose intolerance were more often of Asian origin [15.1% vs. 3.7%, OR 4.64 (1.26–17.12)], had more often a recurrent history of GDM [41.7% vs. 26.7%, OR 3.68 (1.37–9.87)], higher fasting glycaemia (FPG) [5.1 (4.5–5.3) vs. 4.6 (4.3–5.1) mmol/L, OR 1.05 (1.01–1.09)], higher HbA1c [33 (31–36) vs. 32 (30–33) mmol/mol, OR 4.89 (1.61–14.82)], and higher triglycerides [2.2 (1.9–2.8) vs. 2.0 (1.6–2.5) mmol/L, OR 1.00 (1.00–1.01)]. Sensitivity of glucose challenge test (GCT) ≥7.2 mmol/l for glucose intolerance postpartum was 80% (63.1%–91.6%). The area under the curve to predict glucose intolerance was 0.76 (0.65–0.87) for FPG, 0.54 (0.43–0.65) for HbA1c and 0.75 (0.64–0.86) for both combined. In conclusion, nearly one-fifth of women with GDM have glucose intolerance postpartum. A GCT ≥7.2 mmol/L identifies a high risk population for glucose intolerance postpartum.

## 1. Introduction 

Gestational diabetes mellitus (GDM) is defined as diabetes diagnosed in the second or third trimester of pregnancy provided that overt diabetes early in pregnancy has been excluded [1]. Treatment of GDM between 24–28 weeks of pregnancy can reduce adverse pregnancy outcomes, especially large-for-gestational age infants and preeclampsia [2,3]. Women with GDM have a seven-fold increased risk of developing type 2 diabetes (T2DM) later in life compared to normal glucose tolerant (NGT) women during pregnancy [4,5]. Women with persistent glucose intolerance [impaired fasting glucose (IFG) and/or impaired glucose tolerance (IGT)] in early postpartum are a particularly high risk group, with about 50% developing T2DM within 5 years after the delivery [6]. Long-term follow-up is often challenging due to the low attendance rates at screening tests postpartum [7].

The ‘International Association of Diabetes and Pregnancy Study Groups’ (IADPSG) and the World Health Organization (WHO) recommend a universal one-step approach with the 75 g oral glucose tolerance test (OGTT) for screening of GDM with the use of more stringent diagnostic criteria for GDM [8,9]. The IADPSG/2013 WHO criteria are the first diagnostic criteria based on adverse pregnancy outcomes [8,10]. However, data on the risk to develop glucose intolerance postpartum in women with GDM diagnosed by the one-step approach and 2013 WHO criteria, are limited. The use of the 2013 WHO criteria for GDM results in a greater proportion of women diagnosed with mild forms of GDM. This might lead to a lower proportion at risk for postpartum glucose intolerance compared to women diagnosed with GDM by a two-step screening strategy [11,12]. In addition, there are currently few data on the clinical and biochemical risk factors which best predict persistent glucose intolerance in early postpartum in women with GDM diagnosed by a universal one-step screening strategy and the 2013 WHO criteria. More evidence is therefore needed on the risk and predictors for glucose intolerance in early postpartum in women with GDM based on the 2013 WHO criteria. Our aim was to evaluate the prevalence and predictors for glucose intolerance in early postpartum in women with GDM from a large prospective cohort study. 

## 2. Subjects and Methods

The study was registered in ClinicalTrials.gov (NCT02036619). The study protocol was approved by the Institutional Review Boards of all participating centers (B322201420693). Participants provided informed consent before inclusion in the study. 

### 2.1. Study Design

The Belgian Diabetes in Pregnancy study (BEDIP-N) was a multi-centric prospective cohort study that has previously been described in detail [13,14,15]. Women between 18–45 years with singleton pregnancies, and without history of diabetes or bariatric surgery, were recruited between 6–14 weeks of pregnancy [13]. Participants without prediabetes or diabetes in early pregnancy [defined by the American Diabetes Association (ADA) criteria)], received both a non-fasting 50 g glucose challenge test (GCT) and a 75 g 2-h oral glucose challenge test (OGTT) between 24–28 weeks of pregnancy [1,13]. All participants received the OGTT irrespective of the result of the GCT. The diagnosis of GDM was based on the 2013 WHO criteria. We have recently shown that the threshold of the GCT needs to be reduced to at least 7.2 mmol/L, to achieve sensitivity ≥70% to screen for GDM based on the 2013 WHO criteria [14]. The ADA recommended glycemic targets were used for the treatment of GDM [1]. If targets were not achieved with lifestyle measures, insulin therapy was added. Women with GDM were invited for an extra visit 6–16 weeks postpartum to undergo a 75 g OGTT. The ADA criteria were used to define T2DM and glucose intolerance (IFG and/or IGT) [1,13].

### 2.2. Study Assessments

In early pregnancy, baseline characteristics and the obstetrical history were collected [13]. In early pregnancy and at 24–28 weeks of pregnancy, anthropometric measurements were obtained, a clinical examination was performed [weight, body mass index (BMI) and blood pressure] and several self-administered questionnaires were completed [13]. Overweight was defined as a body mass index (BMI) ≥25 Kg/m^2^ and obesity as a BMI ≥30 Kg/m^2^. Excessive weight gain was defined according to the 2009 Institute of Medicine (IOM) guidelines [16]. Early weight gain was calculated as the difference in weight between first prenatal visit and the time of the OGTT, total weight gain was calculated as the difference in weight between first prenatal visit and the delivery. We used a self-designed questionnaire to evaluate breastfeeding [13]. Women were categorized as: almost exclusive breastfeeding (≤45 mL formula feeding/day), half breastfeeding and half formula feeding, and almost exclusive formula feeding (≥150 mL formula feeding/day). 

At first visit between 6–14 weeks of pregnancy, fasting plasma glucose (FPG), fasting insulin, fasting lipid profile (total cholesterol, HDL- and LDL-cholesterol, triglycerides) and HbA1c were measured. The homeostasis model assessment of insulin resistance (HOMA-IR) and β-cell function (HOMA-B), were measured in early pregnancy, as previously described [17]. At the time of the 75 g OGTT during pregnancy and postpartum, a fasting lipid profile and HbA1c were measured. Glucose and insulin were measured fasting, at 30 min, 60 min, and 120 min. For the OGTT, participants had to be fasting for at least 10 h. Increase in triglycerides was defined as the difference between the fasting triglycerides measured in early pregnancy and at the time of the OGTT in pregnancy [13]. 

Different indices of insulin sensitivity [the Matsuda index, a well-established measure of whole-body insulin sensitivity and the homeostasis model assessment of insulin resistance (HOMA-IR), a measure of largely hepatic insulin resistance] and β-cell function [HOMA-B, the insulinogenic index divided by HOMA-IR, Stumvoll index and the insulin secretion-sensitivity index-2 (ISSI-2), an OGTT-derived measure that is analogous to the disposition index obtained from the frequently sampled intravenous glucose tolerance test], were measured, as previously described [13,17,18,19,20,21,22]. The Matsuda and Stumvoll indices were multiplied to calculate the oral disposition index, which assesses β-cell compensation for insulin resistance [23]. 

The analyses of the FPG at 6–14 weeks and the glucose measurements of the OGTT were performed locally at each center. The analyzes of the GCT’s, insulin, lipids and HbA1c levels were performed centrally at the lab of UZ Leuven (Leuven, Belgium) and these results were not communicated to participants and health care providers during the study. Plasma glucose was measured by an automated colorimetric-enzymatic method on a Hitachi/Roche-Modular P analyzer (Basel, Switzerland). Insulin was measured by the immunometric ECLIA (Roche Modular E170). HbA1c was measured by Tosoh Automated Glycohemoglobin Analyzer HLC-723G8. Lipid levels were measured by the immunoassay analyzer Cobas 8000 (Roche, Basel, Switzerland). Coefficients of variance are 1% for glucose, 6% for insulin, about 2% for lipids and 2% for HbA1c in the Lab of UZ Leuven.

### 2.3. Statistical Analysis

Descriptive statistics were given for the two groups where continuous variables are presented as means with standard deviation if normally distributed, or as medians with interquartile range otherwise; categorical variables are presented as frequencies with percentages. Logistic regression models were used for data analysis with postpartum glucose intolerance as a binary response variable and subject characteristics as explanatory variables. Results were reported as odds ratios (OR) with 95% confidence intervals. For variables during pregnancy that were significantly different between women with and without postpartum glucose intolerance, adjusted OR for age and BMI in early pregnancy were analyzed. The discriminative power of continuous variables for postpartum glucose intolerance is presented by receiver operating curve (ROC) curves and estimated as the area under the curve (AUC) with 95% confidence interval. The AUC ranges between 0.5 (discrimination no better than chance) and 1 (perfect discrimination). Diagnostic accuracy of binary or dichotomized variables is estimated as sensitivity, specificity, positive and negative likelihood ratio, and post-test probability, with 95% confidence intervals. A *p*-value <0.05 (two-tailed) was considered significant. Analyses were performed by A. Laenen using SAS software (version 9.4).

## 3. Results

### 3.1. Study Participants

Prospective multicentric cohort study with 1841 women tested for GDM with a 75 g OGTT. GDM was diagnosed in 12.5% (231) of all participants. Of all women with GDM, 83.1% (192) attended the postpartum 75 g OGTT. The OGTT was performed at 14.4 ± 4.1 weeks after delivery. Of all women with an OGTT postpartum, none had T2DM and 18.2% (35) had glucose intolerance postpartum of which 37.1% (13) had IFG, 54.3% (19) had IGT, and 8.6% (3) had IFG and IGT combined.

### 3.2. Characteristics of Women with Glucose Intolerance at the Time of the OGTT Postpartum

Compared to NGT women, women with glucose intolerance postpartum were more insulin resistant [Matsuda index 0.50 (0.33–0.83) vs. 0.74 (0.48–1.08), OR 0.20 (0.06–0.65), *p* = 0.008; HOMA-IR 16.8 (8.9–24.8) vs. 10.2 (7.3–15.6), OR 1.07 (1.02–1.11), *p* = 0.002], had a lower β-cell function [insulinogenic index/HOMA-IR 0.15 (0.11–0.22) vs. 0.26 (0.20–0.36), OR 0.001 (0.00–0.07), *p* = 0.001], lower HDL-cholesterol [1.3 (1.1–1.5) vs. 1.5 (1.3–1.8), OR 0.96 (0.93–0.99), *p* = 0.010], higher fasting triglycerides [1.2 (0.8–2.0) vs. 0.8 (0.6–1.1) mmol/L, OR 1.01 (1.01–1.02), *p* <0.0001] and breastfed less often [66.7% vs. 86.3%, OR 0.32 (0.13–0.75), *p* = 0.009] (Table 1). There was no difference in the duration of breastfeeding or in the rate of exclusive breastfeeding (Table 1). Women with glucose intolerance had a higher BMI compared to NGT women but this was borderline not significant (28.1 ± 6.6 kg/m^2^ vs. 26.1 ± 4.9 kg/m^2^, *p* = 0.050). 

### 3.3. Predictors for Glucose Intolerance Postpartum Based on Characteristics and Biochemical Variables Prepregnancy and during Pregnancy

**Figure 1:**
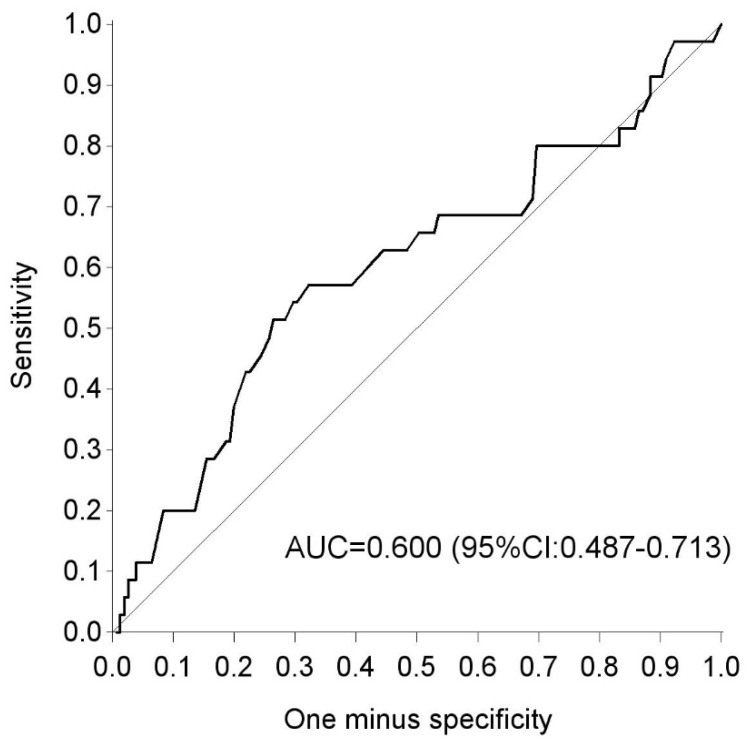
ROC curve for the glucose challenge test in pregnancy as a predictor for glucose intolerance postpartum. ROC: receiver operating curve; AUC: area under the curve.

Based on the characteristics and biochemical variables in early and late pregnancy, compared to NGT women, women with glucose intolerance postpartum had more often an Asian origin [15.1% vs. 3.7%, OR 4.64 (1.26–17.12), *p* = 0.021], were more often multiparous [68.6% vs. 48.4%, OR 2.32 (1.07–5.07), *p* = 0.034], had more often a recurrent history of GDM [41.7% vs. 26.7%, OR 3.68 (1.37– 9.87), *p* = 0.010], a higher fasting glycaemia (FPG) [5.1 (4.5–5.3) vs. 4.6 (4.3–5.1) mmol/L, OR 1.05 (1.01–1.09), *p* = 0.023], a higher HbA1c [33 (31–36) vs. 32 (30–33) mmol/mol, OR 4.89 (1.61–14.82), *p* = 0.005], and higher fasting triglycerides [2.2 (1.9–2.8) vs. 2.0 (1.6–2.5) mmol/L, OR 1.00 (1.00–1.01), *p* = 0.028] at the time of the OGTT during pregnancy (Table 2, Table 3 and Table 4). After adjustment for maternal age and BMI in early pregnancy, a recurrent history of GDM and FPG did not remain significant (Table 3). The AUC on the ROC curve for the 50 g GCT to predict glucose intolerance postpartum was 0.60 (CI 95% 0.449–0.71) (Figure 1). Evaluation of the sensitivity across different GCT thresholds, showed that a GCT ≥ 7.2 mmol/L during pregnancy had the highest sensitivity of 80% (95% CI 63.1%– 91.6%) with a specificity of 26.4% (95% CI 14.6%–27.9%) to predict glucose intolerance postpartum (Table 5). 

### 3.4. Fasting Glycaemia and Hba1c Postpartum to Detect Glucose in Tolerance

**Figure 2:**
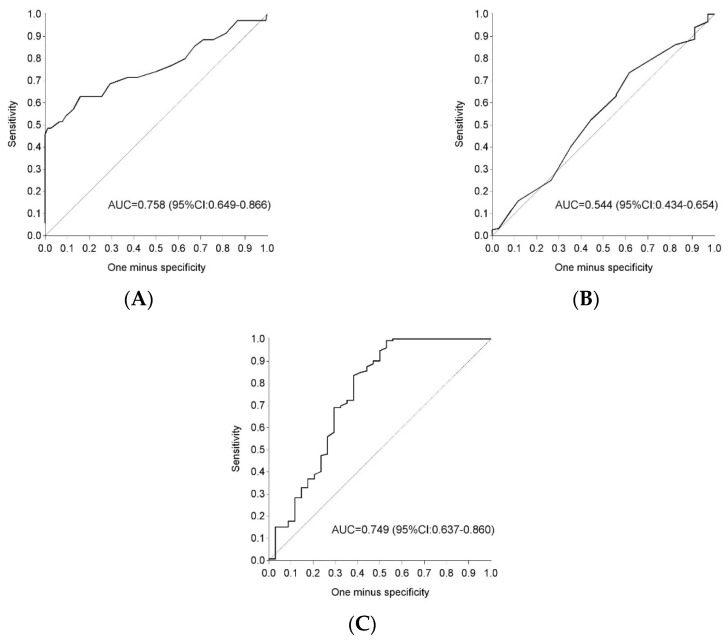
ROC curve for fasting plasma glucose and HbA1c postpartum as a predictor for glucose intolerance. ROC: receiver operating curve; AUC: area under the curve; (**A**) ROC curve for fasting plasma glucose alone; (**B**) ROC curve for HbA1c alone; (**C**) ROC curve for fasting plasma glucose and HbA1c combined.

The AUC for FPG alone in early postpartum to predict glucose intolerance was 0.76 (95% CI 0.65–0.87), the AUC for HbA1c alone was 0.54 (95% CI 0.43–0.65) and the AUC for FPG and Hba1c combined was 0.75 (95% CI 0.64–0.86) (Figure 2).

## 4. Discussion

By diagnosing GDM, a group of women at high risk to develop T2DM and cardiovascular disease later in life was identified [4,5]. Lifestyle interventions and metformin can prevent progression to T2DM on the long-term [24]. However, in normal routine attendance rates at screening tests postpartum are often low, with only 30%–50% of women with recent GDM receiving an OGTT or even a FPG within one year after the delivery and follow-up rates after one year dropping further [25,26]. Even in prospective studies, it remains challenging to obtain high attendance rates as demonstrated in the present study, where nearly one fifth of women with GDM did not attend the OGTT in early postpartum. This is a missed opportunity to timely identify and treat high-risk women for glucose intolerance. A GDM recall register implemented in the northern part of Belgium (Flanders), helps to successfully stimulate screening postpartum in primary care by sending annual reminders to women with a history of GDM [7]. This is in contrast with a South Australian GDM recall register were the response was limited [27]. However, in the South Australian GDM recall register, women who did not respond to a reminder letter were not followed up for a response while the Flemish register did not only use annual reminders by letters and email but if needed, additional reminders by telephone or text messages were used to increase the response rate [7]. Compliance with postpartum screening might also improve if an FPG and/or HbA1c could be used to screen for glucose intolerance instead of the cumbersome OGTT. However, several studies have shown that an FPG or Hba1c alone in early postpartum would miss the majority of women with glucose intolerance [11,28,29]. This is in line with our results showing that the majority of women with glucose intolerance had IGT. IGT is an important risk factor for T2DM, since the highest cumulative incidence to develop T2DM is seen in people with combined IFG/IGT, followed by isolated IGT with the lowest incidence in patients with IFG [30]. Moreover, we show now that combining an FPG and HbA1c in early postpartum, does not improve the accuracy to detect glucose intolerance compared to an FPG alone.

Data on the risk to develop T2DM in women with GDM diagnosed by the one-step approach and 2013 WHO criteria are limited. Before the introduction of the 2013 WHO screening strategy, studies have shown that 30%–50% of women with GDM develop T2DM within the first 10 years after the index pregnancy [4]. The use of the 2013 WHO criteria for GDM results in a greater proportion of women who are diagnosed with mild forms of GDM, which might lead to a lower proportion at risk for postpartum glucose intolerance compared to women diagnosed with GDM by a two-step screening strategy. We show that nearly one-fifth of women with GDM based on a universal one-step screening strategy with the 2013 WHO criteria have glucose intolerance in early postpartum. An Irish study showed a rate of T2DM of 2.2% and prediabetes of 23.7% up to 5 years post-delivery in women with GDM based on the 2013 WHO criteria [12,31]. In contrast, we have previously shown glucose intolerance in 42% three months postpartum in women with GDM diagnosed by a two-step screening strategy with the 2013 WHO criteria [11]. Follow-up of the HAPO study 10–14 years postpartum, showed that untreated women with GDM, defined post hoc by the 2013 WHO criteria, had significantly higher rates of a disorder of glucose metabolism than women without GDM [52.2% vs. 20.1% (T2DM 10.7% vs. 1.6%)] [32]. However, GDM according to the Carpenter & Coustan criteria as defined in the HAPO follow-up study, was associated with a much higher risk for T2DM (20% vs. 7.9%) compared to women with GDM according to the 2013 WHO criteria alone [32]. The high prevalence of glucose intolerance postpartum using the Carpenter & Coustan criteria for GDM is to be expected since these criteria were developed to identify women at high risk for the development of T2DM after the delivery [33]. 

Finding the risk factors for glucose intolerance postpartum after a recent history of GDM is important to identify a subgroup of women at a particularly high risk to progress to T2DM on the long-term. A more intensive follow-up and treatment of this high risk group might be cost-effective to prevent T2DM [34]. The most important risk factors to develop glucose intolerance in early postpartum differ according to the populations studied [35]. A systematic review has shown that BMI, family history of diabetes, non-white ethnicity, advanced maternal age, early diagnosis of GDM, raised FPG and HbA1c, and insulin use during pregnancy are associated with future risk of T2DM [36]. This is in contrast to our study, showing that only an Asian origin, multiparity, a recurrent history of GDM and increased FPG, HbA1c and fasting triglycerides in pregnancy, are risk factors for glucose intolerance in early postpartum. These predictors could be used to identify a subgroup of women with GDM with the highest risk to develop glucose intolerance postpartum and as such help to individualize the intensity of the follow-up. However, other common risk factors such as maternal age and BMI in pregnancy did not emerge as predictors in our study. This might be due to high maternal age of our GDM cohort and the fact that about 50% of GDM women were overweight or obese. Moreover, differences in screening strategy and diagnostic criteria used for GDM between different studies make comparisons difficult. In addition, since women were screened for GDM between 24–28 weeks of pregnancy, we could not evaluate whether testing for GDM before 24 weeks of pregnancy, might identify a group at higher risk for glucose intolerance postpartum.

As can be expected, we show that women with GDM who developed glucose intolerance in early postpartum were more insulin resistant and had an impaired beta-cell function compared to NGT women after delivery. During pregnancy insulin sensitivity and beta-cell dysfunction were not significantly different between both groups. However, women who develop GDM, often already have a subclinical metabolic dysfunction prior to conception compared with NGT women [37]. We speculate that the subgroup of GDM women at the highest risk for T2DM on the long-term, probably already have important predisposing baseline insulin resistance and/or beta-cell dysfunction present before pregnancy. 

In our study, women with glucose intolerance postpartum breastfed less often than NGT women but without a difference in the duration of breastfeeding or in the exclusivity of breastfeeding between both groups. However, the timing of the postpartum OGTT varied between participants and we have no prospective data to evaluate the association between the duration of breastfeeding and risk for glucose intolerance postpartum. In addition, we have no data on breastfeeding beyond three months after delivery. There is now strong evidence that lactation duration (>3–6 months) is independently associated with a graded reduction in the incidence of T2DM [12,38]. This might be due to the lower fasting and postprandial glucose levels and lower insulin secretion seen in lactating women [38]. 

Finally, we show that a GCT can also have a role in predicting postpartum glucose intolerance. We have previously shown that a GCT threshold of 7.2 mmol/L has the best trade-off between sensitivity and specificity to screen for GDM when using the 2013 WHO criteria [14]. Here we show that a GCT threshold of 7.2 mmol/L has also the best sensitivity to predict glucose intolerance in early postpartum. A two-step screening strategy with a GCT has therefore the potential to limit the number of OGTTs to screen for GDM based on the 2013 WHO criteria and at the same time identify a high risk group for glucose intolerance postpartum. 

The strengths of our study are the large prospective multiethnic cohort with the availability of many clinical and biochemical variables in early pregnancy. In addition, we provide data on the predictive value of HbA1c and FPG in early postpartum to detect glucose intolerance. Moreover, we present novel data on the predictive value of a GCT when used in a two-step screening strategy for GDM with the 2013 WHO criteria to predict glucose intolerance postpartum. The limitations are the lack of a control group postpartum and the lack of long-term data postpartum. 

In conclusion, we show that nearly one-fifth of women with GDM based on the 2013 WHO criteria have glucose intolerance in early postpartum and these women have a more adverse metabolic profile. An FPG and HbA1c in early postpartum are not accurate enough to replace a 75 g OGTT to detect glucose intolerance. In addition, a GCT threshold of 7.2 mmol/L has the best sensitivity to predict glucose intolerance in early postpartum. Combining CGT and risk factors identified in the present study could identify a group of women who are at the highest risk for postpartum glucose intolerance and where recall incentives may be most needed. 

## Figures and Tables

**Table 1:** Characteristics of women with glucose intolerance compared to normal glucose tolerant women at the time of the OGTT postpartum.

	Glucose Intolerant Postpartum 18.2% (35)	NGT Postpartum OGTT 81.8% (157)	OR (95% CI)	*p*-Value
Timing postpartum OGTT (weeks)	14.1 ± 4.3	14.5 ± 4.1		0.103
BMI (kg/m^2^)	28.1 ± 6.6	26.1 ± 4.9	1.07 (1.0–1.14)	0.050
% overweight	27.3 (9)	33.1 (51)	1.04 (0.41–2.66)	0.928
% obese	33.3 (11)	19.5 (30)		
Systolic BP (mmHg)	116.4 ± 17.2	116.6 ± 12.7	1.00 (0.97–1.03)	0.937
% Systolic hypertension	6.1 (2)	4.5 (7)	1.35 (0.27–6.84)	0.713
Diastolic BP (mmHg)	74.4 ± 9.5	73.2 ± 9.0	1.01 (0.97–1.06)	0.498
% Diastolic hypertension	6.1 (2)	4.5 (7)	1.35 (0.27–6.84)	0.713
Waist circumference (cm)	92.7 ± 12.5	91.1 ± 12.0	1.01 (0.98–1.04)	0.50
% Waist circumference				
80–88 cm	29.0 (9)	24.7 (36)	2.25 (0.55–9.11)	0.256
>88 cm	61.3 (19)	56.8 (83)	2.06 (0.56–7.50)	0.273
FPG (mmol/L)	5.4 (4.8–5.7)	4.8 (4.5–5.0)	1.16 (1.09–1.23)	**<0.0001**
Glycemia 30 min (mmol/L)	8.4 (7.4–9.2)	7.6 (6.8–8.5)	1.02 (1.01–1.04)	**0.002**
Glycemia 60 min (mmol/L)	8.5 (8.1–9.6)	6.8 (5.7–8.0)	1.03 (1.02–1.05)	**<0.0001**
Glycemia 120 min (mmol/L)	8.0 (6.3–8.9)	5.4 (4.9–6.3)	1.08 (1.05–1.10)	**<0.0001**
HbA1c (%/mmol/mol)	5.3 (5.0–5.5) 34 (31–37)	5.2 (5.0–5.5) 33 (31–37)	1.79 (0.57–5.64)	0.320
Matsuda index	0.50 (0.33–0.83)	0.74 (0.48–1.08)	0.20 (0.06–0.65)	**0.008**
Stumvoll index	162.11 (−523.3;576.7)	−25.01 (−380.7; 274.0)	1.00 (1.00–1.00)	0.218
Oral disposition index	64.3 (−382.1;218.3)	−18.1 (−381.0;157.1)	1.00 (1.00–1.00)	0.393
HOMA-IR	16.8 (8.9–24.8)	10.2 (7.3–15.6)	1.07 (1.02–1.11)	**0.002**
HOMA-B	681.7 (466.7–1163.1)	784.0 (561.6–1210.8)	1.00 (1.00–1.00)	0.683
ISSI-2	0.14 (0.08–0.49)	0.25 (0.12–0.45)	0.56 (0.19–1.67)	0.298
Insulinogenic index/HOMA-IR	0.15 (0.11–0.22)	0.26 (0.20–0.36)	0.001 (0.00–0.07)	**0.001**
Total cholesterol (mmol/L)	4.6 (4.2–5.3)	4.8 (4.1–5.2)	1.00 (0.99–1.02)	0.587
HDL-cholesterol (mmol/L)	1.3 (1.1–1.5)	1.5 (1.3–1.8)	0.96 (0.93–0.99)	**0.010**
LDL-cholesterol (mmol/L)	2.4 (2.2–3.3)	2.7 (2.1–3.2)	1.00 (0.99–1.01)	0.845
Triglycerides (mmol/L)	1.2 (0.8–2.0)	0.8 (0.6–1.1)	1.01 (1.01–1.02)	**<0.0001**
% Breastfeeding	66.7 (22)	86.3 (132)	0.32 (0.13–0.75)	**0.009**
Duration breastfeeding				
1 month	18.2 (4)	8.7 (11)	0.65 (0.35–1.20)	0.170
2 months	18.2 (4)	15.9 (20)		
3 months	63.6 (14)	75.4 (95)		
Intensity breastfeeding				
% mostly exclusive breastfeeding	71.4 (10)	71.7 (71)		
% half breastfeeding and half formula feeding	14.3 (2)	14.1 (14)		
% mostly formula breastfeeding	7.1 (1)	7.1 (7)		
Mostly exclusive breastfeeding vs. half breastfeeding and half formula feeding Mostly exclusive breastfeeding vs. mostly exclusive formula feeding			1.01 (0.200–5.14) 1.01 (0.11–9.13)	0.986 0.990

OGTT: 75 g oral glucose tolerance test; OR: odds ratio; CI: confidence interval; NGT: normal glucose tolerance; Categorical variables are presented as frequencies % (n); continuous variables are presented as mean ± SD if normally distributed and as median ± IQR if not normally distributed; Logistic regression models were used for data analysis with postpartum glucose intolerance as a binary response variable and subject characteristics as explanatory variables; overweight: BMI ≥25– 29.9 Kg/m^2^; obesity: BMI ≥30 Kg/m^2^; hypertension: blood pressure systolic ≥140 mmHg or diastolic ≥90 mmHg; PCOS: polycystic ovarian syndrome; A history of GDM and a history of a macrosomic baby (>4 Kg) were calculated on the number of women with a previous pregnancy; HOMA-IR: homeostatic model assessment of insulin resistance; HOMA-B: homeostatic model assessment of beta-cell function; ISSI-2: the insulin secretion-sensitivity index-2; Differences are considered significant at *p*-value <0.05.

**Table 2:** Predictors of glucose intolerance postpartum based on general characteristics and prepregnancy risk factors.

	Glucose Intolerant Postpartum 18.2% (35)	NGT Postpartum OGTT 81.8% (157)	OR (95% CI)	*p*-Value	Adjusted OR (95% CI)	*p*-Value
Age (years)	32.7 ± 4.4	32.2 ± 4.7	1.02 (0.94–1.10)	0.595		
% Non-Caucasian	20.0 (7)	16.7 (26)	1.25 (0.49–3.16)	0.638		
% Northern-African	3.4 (1)	4.4 (6)	0.77 (0.09–6.68)	0.816		
% Asian	15.1 (5)	3.7 (5)	**4.64 (1.26–17.12)**	**0.021**	**5.10 (1.34–19.37)**	**0.017**
Prepregnancy BMI (kg/m^2^)	26.9 ± 6.7	25.6 ± 5.4	1.04 (0.97–1.11)	0.256		
% overweight prepregnancy	28.1 (9)	24.7 (37)	1.56 (0.60–4.03)	0.358		
% obese prepregnancy	28.1 (9)	20.7 (31)	1.70 (0.67–4.33)	0.265		
% highest degree primary school	2.9 (1)	1.4 (2)	2.24 (0.20–25.51)	0.515		
% highest degree lower secondary school	5.9 (2)	3.4 (5)	1.79 (0.33–9.68)	0.497		
% no education higher than secondary school	18.2 (6)	23.2 (33)	0.73 (0.28–1.93)	0.531		
% no paid job	11.8 (4)	7.1 (11)	1.73 (0.52–5.81)	0.373		
% income from benefits	2.9 (1)	1.9 (3)	1.53 (0.15–15.23)	0.714		

OR: odds ratio, adjusted OR (odds ratio) for maternal age and BMI in early pregnancy; CI: confidence interval; NGT: normal glucose tolerance; Categorical variables are presented as frequencies % (n); continuous variables are presented as mean ± SD if normally distributed and as median ± IQR if not normally distributed; Logistic regression models were used for data analysis with postpartum glucose intolerance as a binary response variable and subject characteristics as explanatory variables; Differences are considered significant at *p*-value <0.05.

**Table 3:** Predictors of glucose intolerance postpartum based on clinical and biochemical risk factors 6–16 weeks of pregnancy.

	Glucose Intolerant Postpartum 18.2% (35)	NGT Postpartum OGTT 81.8% (157)	OR (95% CI)	*p*-Value	Adjusted OR (95% CI)	*p*-Value
BMI (kg/m^2^)	27.3 ± 6.5	26.4 ± 5.1	1.03 (0.96–1.10)	0.380		
% overweight	28.6 (10)	27.6 (43)	1.18 (0.49–2.85)	0.716		
% obese	28.6 (10)	23.1 (36)	1.43 (0.58–3.48)	0.436		
Systolic BP (mmHg)	114.9 ± 13.9	117.2 ± 11.0	0.98 (0.95–1.0)	0.284		
% Systolic hypertension	8.6 (3)	1.9 (3)	4.78 (0.92–24.77)	0.062		
Diastolic BP (mmHg)	70.8 ± 8.9	72.5 ± 8.9	0.98 (0.94–1.02)	0.324		
% Diastolic hypertension	5.7 (2)	3.2 (5)	1.83 (0.34–9.84)	0.481		
Waist circumference (cm)	92.0 ± 14.1	90.3 ± 12.8	1.01 (0.98–1.04)	0.512		
% waist circumference						
80–88 cm	34.4 (11)	31.8 (48)	1.42 (0.45–4.48)	0.549		
>88 cm	50.0 (16)	47.7 (72)	1.38 (0.46–4.09)	0.564		
% smoking before pregnancy	8.6 (3)	3.2 (5)	1.73 (0.82–3.65)	0.147		
% smoking during pregnancy	45.7 (16)	32.7 (51)	2.83 (0.64–12.45)	0.168		
% multiparity	68.6 (24)	48.4 (76)	**2.32 (1.07–5.07)**	**0.034**	**2.37 (1.06–5.31)**	**0.036**
% history of miscarriage	40.0 (14)	29.9 (47)	1.56 (0.73–3.33)	0.250		
% history of GDM	41.7 (10/24)	26.7 (20/75)	**3.68 (1.37–9.87)**	**0.010**	1.89 (0.72–4.97)	0.197
% first degree family history of GDM	6.2 (2)	6.2 (9)	0.98 (0.20–4.79)	0.985		
% first degree family history of diabetes	25.7 (9)	16.4 (25)	1.71 (0.68–4.25)	0.251		
% history of macrosomia	12.5 (3/24)	15.8 (13/76)	1.70 (0.42–6.92)	0.459		
% history impaired glucose tolerance	9.4 (3)	2.2 (3)	0.22 (0.04–1.13)	0.069		
% history of PCOS	2.9 (1)	5.7 (9)	2.07 (0.25–16.87)	0.498		
% fertility treatment	25.7 (9)	17.2 (27)	0.60 (0.25–1.42)	0.246		
FPG (mmol/L)	4.7 (4.5–5.0)	4.7 (3.4–4.9)	0.10 (0.95–1.05)	0.928		
% FPG ≥ 5.1 mmol/L	11.8 (4)	13.5 (21)	1.17 (0.37–3.64)	0.791		
HbA1c (%/mmol/mol)	5.1 (4.9–5.3) 32 (30–34)	5.0 (4.9–5.3) 31 (30–34)	0.734 (0.23–2.33)	0.599		
HOMA-IR	10.2 (7.2–17.6)	10.7 (7.6–16.9)	1.00 (0.96–1.05)	0.839		
HOMA-B	830.2 (630.0–1284.0)	983.5 (667.2–1393.6)	0.10 (0.10–1.00)	0.211		
Total cholesterol (mmol/L)	4.5 (4.1–5.7)	4.8 (4.2–5.4)	1.00 (0.99–1.01)	0.805		
HDL-cholesterol (mmol/L)	1.7 (1.3–1.9)	1.7 (1.5–2.0)	1.02 (0.99–1.05)	0.197		
LDL-cholesterol (mmol/L)	2.2 (1.9–3.1)	2.4 (2.1–2.9)	1.00 (0.99–1.01)	0.887		
Triglycerides (mmol/L)	1.2 (0.9–1.7)	1.1 (0.9–1.3)	0.99 (0.99–1.00)	0.140		

OR: odds ratio, adjusted OR (odds ratio) for maternal age and BMI in early pregnancy; CI: confidence interval; NGT: normal glucose tolerance; Categorical variables are presented as frequencies % (n); continuous variables are presented as mean ± SD if normally distributed and as median ± IQR if not normally distributed; Logistic regression models were used for data analysis with postpartum glucose intolerance as a binary response variable and subject characteristics as explanatory variables; overweight: BMI ≥25–29.9 Kg/m^2^; obesity: BMI ≥30 Kg/m^2^; hypertension: blood pressure systolic ≥140 mmHg or diastolic ≥90 mmHg; PCOS: polycystic ovarian syndrome; A history of GDM and a history of a macrosomic baby (>4 Kg) were calculated on the number of women with a previous pregnancy; HOMA-IR: homeostatic model assessment of insulin resistance; HOMA-B: homeostatic model assessment of beta-cell function; ISSI-2: the insulin secretion-sensitivity index-2; Differences are considered significant at *p*-value <0.05.

**Table 4:** Predictors of glucose intolerance postpartum based on clinical and biochemical risk factors 24–28 weeks of pregnancy.

	Glucose Intolerant Postpartum 18.2% (35)	NGT Postpartum OGTT 81.8% (157)	OR (95% CI)	*p*-Value	Adjusted OR (95% CI)	*p*-Value
BMI (kg/m^2^)	30.0 ± 6.6	28.9 ± 4.8	1.04 (0.97–1.11)	0.268		
% overweight	38.2 (13)	41.3 (62)	0.92 (0.35–2.43)	0.862		
% obese	38.2 (13)	35.3 (53)	1.07 (0.40–2.85)	0.888		
Systolic BP (mmHg)	115.3 ± 11.8	114.7 ± 11.2	1.00 (0.97–1.04)	0.767		
% Systolic hypertension	5.7 (2)	2.6 (4)	2.30 (0.40–13.10)	0.347		
Diastolic BP (mmHg)	69.7 ± 7.1	68.7 ± 8.4	1.01 (0.97–1.06)	0.517		
% Diastolic hypertension	0.0 (0)	2.6 (4)	0.00	0.980		
Fasting plasma glycemia (mmol/L)	5.1 (4.5–5.3)	4.6 (4.3–5.1)	**1.05 (1.01–1.09)**	**0.023**	1.04 (1.00–1.09)	0.070
Glycemia 30 min (mmol/L)	8.5 (7.1–9.6)	8.1 (7.4–8.9)	1.01 (0.10–1.03)	0.128		
Glycemia 60 min (mmol/L)	9.7 (8.3–10.5)	9.6 (8.7–10.3)	1.00 (0.98–1.01)	0.974		
Glycemia 120 min (mmol/L)	8.8 (8.3–8.9)	8.6 (7.7–9.1)	1.01 (0.99–1.02)	0.196		
HbA1c (%/mmol/mol)	5.2 (5.0–5.4) 33 (31–36)	5.1 (4.9–5.2) 32 (30–33)	**4.89 (1.61–14.82)**	**0.005**	**4.43** **(1.37–14.35)**	**0.013**
% GCT ≥7.2 mmol/L	80.00 (28)	73.5 (114)	1.44 (0.58–3.54)	0.430		
Matsuda index	0.30 (0.21–0.50)	0.40 (0.25–0.52)	0.40 (0.06–2.69)	0.349		
Stumvoll index	529.3 (32.5–1167.8)	460.1 (20.5–1154.4)	1.00 (1.00–1.00)	0.503		
Oral disposition index	150.7 (12.7–274.8)	186.1 (10.6–289.6)	1.00 (0.10–1.00)	0.421		
HOMA-IR	20.4 (13.4–30.5)	16.3 (11.1–26.5)	1.01 (0.98–1.03)	0.520		
HOMA-B	1376.3 (947.6–2047.1)	1407.5 (1055.1–2198.2)	1.00 (1.00–1.00)	0.429		
ISSI-2	0.07 (0.05–0.17)	0.10 (0.04–0.17)	0.96 (0.04–21.14)	0.979		
Insulinogenic index/HOMA-IR	0.18 (0.12–0.25)	0.21 (0.16–0.31)	0.07 (0.00–2.32)	0.137		
Total cholesterol (mg/dl)	5.9 (5.4–6.9)	6.3 (5.7–7.0)	0.99 (0.99–1.00)	0.296		
HDL-cholesterol (mmol/L)	1.9 (1.5–2.2)	1.9 (1.6–2.2)	0.99 (0.97–1.02)	0.559		
LDL-cholesterol (mmol/L)	3.1 (2.6–3.7)	3.5 (2.9–4.9)	0.99 (0.98–1.00)	0.055		
Triglycerides (mmol/L)	2.2 (1.9–2.8)	2.0 (1.6–2.5)	**1.00 (1.00–1.01)**	**0.028**	**1.00 (1.00–1.01)**	**0.049**
Triglycerides change (mmol/L)	0.9 (0.6–1.5)	0.9 (0.6–1.2)	1.01 (1.00–1.01)	0.082		
Gestational weight gain (Kg)	9.1 ± 5.1	8.1 ±4.7	1.04 (0.96–1.13)	0.299		
% excessive gestational weight	20.6 (7)	16.3 (22)	0.99 (0.33–0.93)	0.982		
Gestational age diagnosis GDM (weeks)	26.7 ±1.0	26.9 ± 1.1	0.78 (0.56–1.08)	0.139		
% insulin treatment	25.7 (9)	13.4 (21)		0.281		
% Long-acting	5.7 (2)	2.5 (4)	2.61 (0.45–15.03)	0.281		
% short acting	8.6 (3)	6.4 (10)	1.57 (0.40–6.09)	0.515		
% short-and long acting	11.4 (4)	4.7 (7)	2.10 (0.82–10.95)	0.098		
Number of insulin injections						
1 2 3 4	22.2 (2) 11.1 (1) 33.3 (3) 33.3 (3)	33.3 (7) 14.3 (3) 28.6 (6) 23.8 (5)	1.29 (0.65–2.56)	0.459		
Gestational age at start insulin (weeks)	28.6 ± 2.6	30.4 ± 2.2	0.67 (0.43–1.04)	0.074		
Total dose insulin (units)	22.0 ± 15.4	15.6 ±12.1	1.05 (0.98–1.12)	0.132		

OR: odds ratio, adjusted OR (odds ratio) for maternal age and BMI in early pregnancy; CI: confidence interval; NGT: normal glucose tolerance; Categorical variables are presented as frequencies % (n); continuous variables are presented as mean ±SD if normally distributed and as median ± IQR if not normally distributed; Logistic regression models were used for data analysis with postpartum glucose intolerance as a binary response variable and subject characteristics as explanatory variables; overweight: BMI ≥25–29.9 Kg/m^2^; obesity: BMI ≥30 Kg/m^2^; hypertension: blood pressure systolic ≥140 mmHg or diastolic ≥90 mmHg; HOMA-IR: homeostatic model assessment of insulin resistance; HOMA-B: homeostatic model assessment of beta-cell function; ISSI-2: the insulin secretion-sensitivity index-2; Differences are considered significant at *p*-value <0.05.

**Table 5:** Sensitivity and specificity of the glucose challenge test for glucose intolerance postpartum.

Threshold GCT	Sensitivity	Specificity	LR+	LR–	Positive Post-test Probability	Negative Post-test Probability
	**% (95% CI)**	**% (95% CI)**	**(95% CI)**	**(95% CI)**	**(95% CI)**	**(95% CI)**
	**n/N**	**n/N**				
≥7.8 mmol/L	68.6	41.3	1.2	0.76	20.6%	14.5%
	(50.7–83.1)	(33.4–49.5)	(0.9–1.5)	(0.45–1.29)	(12.9–29.4)	(8.9–21.5)
	24/35	64/155				
≥7.5 mmol/L	68.6	32.9	1.0	0.96	18.5%	17.5%
	(50.7–83.1)	(25.6–40.9)	(0.8–1.3)	(0.56–1.64)	(11.7–26.6)	(10.8–25.5)
	24/35	51/155				
≥7.2 mmol/L	80.0	26.4	1.1	0.76	19.5%	14.4%
	(63.1–91.6)	(19.7–34.1)	(0.9–1.3)	(0.37–1.54)	(12.3–27.9)	(8.4–21.7)
	28/35	41/155				
≥6.9 mmol/L	80.0	20.6	1.0	0.97	18.3%	17.7%
	(63.1–91.6)	(14.6–27.9)	(0.8–1.2)	(0.47–2.01)	(11.6–26.3)	(10.6–26.1)
	28/35	32/155				
≥6.7 mmol/L	80.0	16.8	1.0	1.19	17.6%	21.0%
	(63.1–91.6)	(11.3–23.6)	(0.8–1.1)	(0.56–2.52)	(11.2–25.3)	(12.6–30.4)
	28/35	26/155				

GCT: 50 g glucose challenge test; CI: confidence interval; Sensitivity: n = number with GCT ≥ cut-off; N = number with glucose intolerance postpartum; Specificity: n = number with GCT < cut-off; N = number without glucose intolerance postpartum; LR+: positive likelihood ratio; LR–: negative likelihood ratio.
